# Early-Stage Growth Mechanism and Synthesis Conditions-Dependent Morphology of Nanocrystalline Bi Films Electrodeposited from Perchlorate Electrolyte

**DOI:** 10.3390/nano10061245

**Published:** 2020-06-27

**Authors:** Daria Tishkevich, Sergey Grabchikov, Tatiana Zubar, Denis Vasin, Sergei Trukhanov, Alla Vorobjova, Dmitry Yakimchuk, Artem Kozlovskiy, Maxim Zdorovets, Sholpan Giniyatova, Dmitriy Shimanovich, Dmitry Lyakhov, Dominik Michels, Mengge Dong, Svetlana Gudkova, Alex Trukhanov

**Affiliations:** 1 Laboratory of Magnetic Films Physics, Cryogenic Research Department, Scientific-Practical Materials Research Centre of National Academy of Sciences of Belarus, 220072 Minsk, Belarus; dashachushkova@gmail.com (D.T.); gss@physics.by (S.G.); fix.tatyana@gmail.com (T.Z.); hisrb@mail.ru (D.V.); sv_truhanov@mail.ru (S.T.); yakimchuk@physics.by (D.Y.); 2 Laboratory of Single Crystal Growth, South Ural State University, 454080 Chelyabinsk, Russia; svetlanagudkova@yandex.ru; 3 Department of Micro- and Nanoelectronics, Belarusian State University of Informatics and Radioelectronics, 220013 Minsk, Belarus; vorobjova@bsuir.by (A.V.); shdl@tut.by (D.S.); 4 Engineering Profile Laboratory, L.N. Gumilyov Eurasian National University, Nur-Sultan 010008, Kazakhstan; kozlovskiy.a@inp.kz (A.K.); mzdorovets@inp.kz (M.Z.); giniyatova_shg@enu.kz (S.G.); 5 Laboratory of Solid State Physics, The Institute of Nuclear Physics, Almaty 050032, Kazakhstan; 6 Department of Intelligent Information Technologies, Ural Federal University Named after the First President of Russia B.N. Yeltsin, 620075 Yekaterinburg, Russia; 7 Computer, Electrical and Mathematical Science and Engineering Division, 4700 King Abdullah University of Science and Technology, Thuwal 23955-6900, Saudi Arabia; dmitry.lyakhov@kaust.edu.sa (D.L.); dominik.michels@kaust.edu.sa (D.M.); 8 Department of Resource and Environment, Northeastern University, Shenyang 110819, China; mg_dong@163.com; 9 Moscow Institute of Physics and Technology (State University), 141701 Dolgoprudny, Russia

**Keywords:** bismuth, lead, films, electrochemical deposition, co-deposition, growth mechanism, morphology, perchlorate electrolyte

## Abstract

Bi nanocrystalline films were formed from perchlorate electrolyte (PE) on Cu substrate via electrochemical deposition with different duration and current densities. The microstructural, morphological properties, and elemental composition were studied using scanning electron microscopy (SEM), atomic force microscopy (AFM), and energy-dispersive X-ray microanalysis (EDX). The optimal range of current densities for Bi electrodeposition in PE using polarization measurements was demonstrated. For the first time, it was shown and explained why, with a deposition duration of 1 s, co-deposition of Pb and Bi occurs. The correlation between synthesis conditions and chemical composition and microstructure for Bi films was discussed. The analysis of the microstructure evolution revealed the changing mechanism of the films’ growth from pillar-like (for Pb-rich phase) to layered granular form (for Bi) with deposition duration rising. This abnormal behavior is explained by the appearance of a strong Bi growth texture and coalescence effects. The investigations of porosity showed that Bi films have a closely-packed microstructure. The main stages and the growth mechanism of Bi films in the galvanostatic regime in PE with a deposition duration of 1–30 s are proposed.

## 1. Introduction

Bismuth and its alloys are interesting materials generally used in various applications. This chemical element has highly unusual physical properties including thermoelectrical, electronic, and magnetic ones [1,2,3,4]. It is a semimetal with a high anisotropic Fermi surface, and low and very large carrier densities and mobilities, respectively [5]. For example, nanostructured thin Bi films revealed exotic magneto-electronic properties which make them appealing materials for spintronic applications [6,7,8], displayed large positive magnetoresistance [9], and Bi-modified electrodes due to their catalytic properties and mechanical stability were used for electrochemical sensors instead of poisonous mercury electrodes [10,11,12,13,14]. From the point of view of condensed matter research and other significant applications, especially in the films form, have been applied to test several quantum confinement phenomena [5,15,16,17]. Bi-based sensitive radiation detectors, such as microcalorimeters demonstrate good X-ray absorption because of Bi’s large atomic number and very low heat capacity [5,18,19,20,21]. Uses of Bi-based composites and coatings offer a very applicable alternative to the lead protection against proton radiation due to much more environmentally friendly Bi [22,23]. Bi_2_O_3_-glasses showed high gamma radiation effectiveness [24], and textiles with filled Bi_2_O_3_ particles are applied in the manufacture of overalls for medical personnel working on gamma-ray systems [25]. Multilayer structures with alternating layers light (Ba, Sb)/heavy (Bi, W) elements on the polymer substrate provide the protection equivalent to the lead materials, but with 25% lower mass-dimensional parameters [26]. Bi and Bi/Bi_2_O_3_ surfaces with dendritic microstructure demonstrated superhydrophobic properties [27,28].

Electrochemical deposition is one of the most promising methods for the thin and thick films synthesis. It provides advantages such as low manufacturing costs, low synthesis temperatures, a high rate growth, and a high level of pure and homogeneous films. Besides, electrodeposition enables the films’ stoichiometry, thickness, and microstructure, which can be controlled by deposition conditions variation. It is possible to obtain films with different properties and functions by changing the parameters, such as current density, electrolyte composition, and substrate type. A large number of materials can be obtained in this way [29,30,31]. It has been shown that Bi can be produced in different configurations such as dense or particulated films [22,32] and sparse particles (hexagons, dendrites, rods, wires, etc.) [14,27,33]. Bi nanowires, imbedded in Al_2_O_3_ matrix, showed their potential use for enhancing thermoelectric performance [34,35,36,37,38,39,40]. Many authors have focused on the Bi deposition onto noble metals (evaporated Au and polycrystalline Cu) and non-metallic substrates, such as semiconductors [41,42] and glassy carbon [43]. However, the number of authors who produce bismuth films onto metallic substrates is very limited [23,44,45]. Many previous studies have been dedicated to Bi films electrodeposition from lactate, tartrate, silicate, nitrate, citric, hydrochloric, stearic, sulfate, and pyrophosphate electrolytes [13,27,32,41,46,47,48,49,50]. However, the literature on the perchlorate electrolytes study is not comprehensive enough [51,52]. This electrolyte has several advantages: Homogeneous and dense coatings obtaining, high deposition rates, and it also approximates 100% current efficiency. Thick coating obtaining is one of the main tasks in the radiation protective shields manufacturing, so the use of the electrolyte, which allows achieving high electrodeposition rates, is very relevant [22,23]. To maximize the protective efficiency against various ionizing radiations is essential for semiconductor shielding applications as well as to get a deeper understanding of the Bi growth controllability [53,54]. Moreover, the investigation on the initial stage of Bi electrodeposition in perchlorate electrolyte, including the nucleation processes and its growth mechanism, is extremely insufficient. Consequently, an intensive understanding of nucleus formation and growth during Bi electrodeposition is critical for good quality Bi films obtaining.

In this paper, we reported the electrodeposition of Bi onto Cu substrate from perchlorate electrolyte in the galvanostatic regime, and its nucleation and growth mechanism were investigated. These studies contribute to a more detailed understanding of the fine-grained and dense films obtaining process as a promising material for many practical applications. 

## 2. Materials and Methods 

Experimental samples were thin Bi films electrodeposited onto Cu substrate (thickness 70 µm). The copper substrates were treated in a Viennese lime mixture, consisting of CaO and MgO (1v:1v). Then, the degreased surface of the copper substrates was polished in the ammonium persulfate solution (120 g·L^−1^ (NH_4_)_2_S_2_O_8_ + 20 g·L^−1^ H_2_SO_4_) within 30 s at room temperature for the removal of an oxide film from the copper surface. After treatment of the Cu substrates, the surface roughness was 9.6 nm. Bi electrochemical deposition was carried out from the perchlorate electrolyte (PE) in the galvanostatic regime under the following conditions and solution compositions: Bi_2_O_3_—40 g·L^−1^, concentrated 65% HClO_4_—400 mL·L^−1^, distilled H_2_O—up to 1 L, temperature—20–25 °C, and current density—10–20 mA/cm^2^. All chemicals used were commercial reagents with analytical purity. Bismuth rods with 8 mm diameter, containing 97.5% Bi and up to 2.5% PbO_2_, were used as anodes. The ratio of the bismuth anodes’ area to the cathodes’ area was in the range from 0.3 to 0.5. This ratio is necessary to ensure the mode of electropolishing of anodes, which leads to their more uniform dissolution. The Bi electrodeposition was performed using a power source B5-78/6 coupled with a control device (to control the deposition duration). The duration of electrodeposition was varied from 1 to 30 s. All experiments were carried out without mixing due to the short deposition duration. The deposition process in PE was investigated via voltammetric technique using the Autolab PGSTAT302N potentiostat/galvanostat. The measurements were carried out in a three-electrode cell with an Ag/AgCl reference electrode. 

Bi films surface morphology study was realized with the scanning electron microscope (SEM) JEOL JSM-7500F at an accelerating voltage of 5 kV and with the atomic force microscope (AFM) NT-206 in a contact scanning mode. The AFM measurements were carried out for at list five points on the surface area of each sample. A standard V-shaped silicon probe with a 3 N∙m^−1^ stiffness coefficient and a 5 nm tip curvature radius were used. The porosity was evaluated using custom software, which processed the SEM images and then allowed to obtain contrast images of porosity. The calculation of the porosity value was determined using the following formula:

(1) $$P=\frac{S_{P}}{S_{g}}\cdot 100\%$$

where S_p_—pores projection area and S_g_—grains projection area.

Gwyddion software package was used to analyze the grain size, and then statistic grain analysis was performed. The standard method was applied which is described in detail in [55]. The thickness of the deposited films was determined by the stepwise abrasion using a diamond probe with the nanoindenter Hysitron TI 750 Ubi [56].

The chemical composition of the samples was evaluated by energy-dispersive X-ray microanalysis (EDX) using a Bruker XFlash MIN SVE microanalyzer (Bruker Nano GmbH, Berlin, Germany), working in conjunction with a Hitachi TM3030 SEM (Hitachi Group, Tokyo, Japan). The accelerating voltage during EDX experiments was 9–14 keV. 

## 3. Results and Discussion

### 3.1. Polarization Measurements

**Figure 1:**
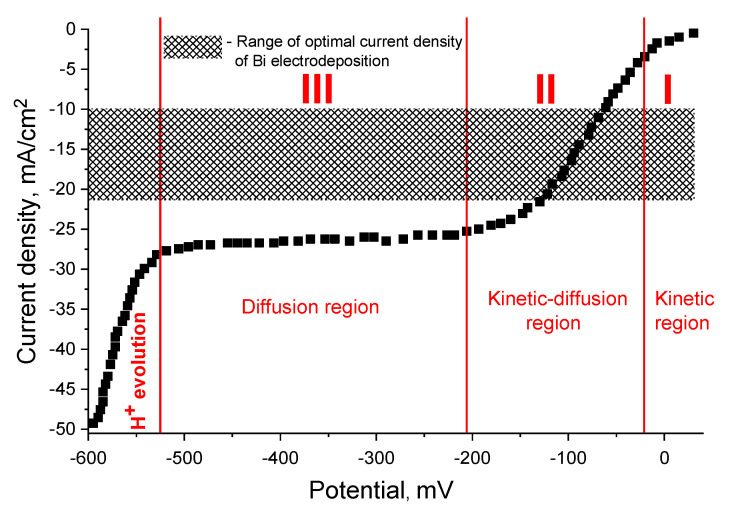
The polarization curve of the Bi electrodeposition from perchlorate electrolyte (PE) without mixing.

Polarization measurements were carried out in a PE, without mixing, to determine the working (optimal) range of current densities and to identify the optimal modes for the deposition of Bi films (Figure 1). Bismuth electrodeposition begins at potentials slightly more positive than 0 V relative to the Ag/AgCl reference electrode, namely, at a potential of 32 mV, which is close to the equilibrium potential of the Bi electrode in this solution.

Three main regions can be observed on the polarization curve that describes the electrochemical and diffusion kinetics of the electrodeposition process (Figure 1). Initially, at small deviations of the potential from zero, the deposition of Bi proceeds in the kinetic regime (region I of Figure 1). In this case, as the cathodic polarization increases, the reaction rate rises, and the diffusion begins to slow it down without supplying a sufficient number of ions to the electrode surface per unit time. A region of mixed kinetic-diffusion processes arises (region II in Figure 1). This inhibition is more and more affected as the cathodic polarization increases and, therefore, the rate of the electrode reaction is increasingly limited by the diffusion. This can be observed in the potential range from −205 to −525 mV (diffusion plateau—region III). Then, the conditions of the limiting current come when the diffusion rate reaches the highest possible value.

Therefore, the curve is parallel to the abscissa axis, which indicates the impossibility of changing the reaction rate by increasing the polarization. At potential values above −525 mV, the cathode current again begins to increase sharply, which is associated with the deposition of a dark powdery precipitate on the cathode surface. The presence of this region is associated with the onset of H^+^ evolution on the cathode surface, and the evolution becomes more intense as the potential increases. 

In this regard, to study the influence of synthesis conditions (deposition modes), namely, current density (*D_c_*) and deposition duration (*t*) on the morphology and microstructure of Bi films, we selected a range of *D_c_* from 10 to 20 mA/cm^2^ without mixing, which is in the range of optimal current densities (marked with hatching lines in Figure 1). 

As a result, three series of samples were obtained with different synthesis conditions. Table 1 includes data on the main samples described in the article. However, for the statistical evaluation of the influence of synthesis conditions on the Bi electrodeposition, a larger number of samples were prepared. The description of the main experimental samples and their characteristics is presented in Table 1.

### 3.2. Chemical Composition Study

The EDX analysis of the experimental samples showed that the electrodeposited films of all series contain such components as Bi, Pb, Cu, O, and C. The oxygen and carbon content in the samples is at the background level. The presence of the copper phase is explained by the usage of Cu for the deposition of Bi and the duration in the range from units to tens of seconds, which contributes to the formation of a very thin film on the substrate surface. Moreover, an interesting behavior of the two main phases of Pb and Bi in the films was noted. The presence of these phases depends on both the deposition duration and the value of current density. For a more detailed study of such an anomaly, the relative percentage concentration (C) of the two main phases (we called them “Pb-rich” and “Bi-rich”) was calculated for each series of samples. The calculations did not take into account the content of other chemical elements (O, C, Cu). The calculations were performed using the equation:

(2) $$C=\frac{C_{Bi}\;\left(C_{Pb}\right)}{C_{Bi}+C_{Pb}}\cdot 100\%$$

where *C_Bi_*—Bi content in the sample, atom. % and *C_P__b_*—Pb content in the sample, atom. %. 

**Figure 2:**
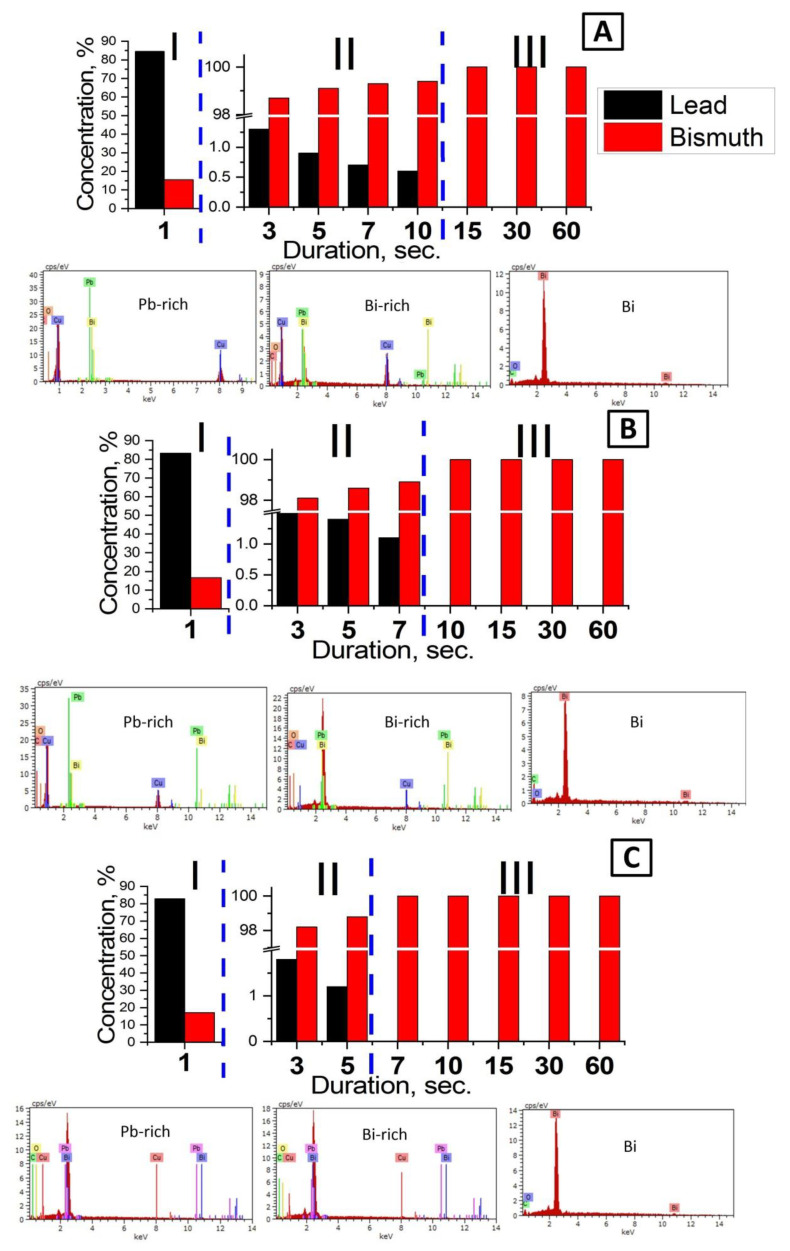
The dependence of the Pb and Bi concentration in electrodeposited films on the deposition duration at current densities of 10 mA/cm^2^ (**A**), 15 mA/cm^2^ (**B**), and 20 mA/cm^2^ (**C**). The insets show the data of energy-dispersive X-ray microanalysis (EDX) analysis.

The study of the chemical composition (Figure 2) showed that for all ranges of current densities, there are three regions: Region I—Pb-rich phase with Pb concentration up to 84.5% (samples 1_10, 1_15, and 1_20); region II—Bi-rich phase with small amount of Pb (up to 1.9%)—samples 2.10, 2,15, and 2_20; and region III—pure Bi phase (other samples of Table 1). In the early stages an abnormal co-deposition of Pb and Bi occurred due to Pb being thermodynamically less metal and deposits preferentially than Bi. Unfortunately, the observation of such Pb and Bi co-deposition has never been mentioned in the scientific literature (the data is absent). Firstly, the researchers were probably not studying the electrochemical deposition of Bi from PE and the nucleation processes in the initial stages. Secondly, the researchers were supposedly using pure Bi anodes for electrodeposition. However, in our case, the presence of the Pb impurity in the films is due to its existence in the original Bi rods, which were used as anodes. In electrochemistry, such anodes are commonly used to deposit Bi films, since Bi is often produced by processing polymetallic lead concentrates, where the presence of a small amount of Pb impurities in Bi (up to 3%) is acceptable [57]. 

An increase in the *D_c_* to 20 mA/cm^2^ (samples 1_10–1_20) decreases the ratio of the Pb-rich phase from Pb_84.5_:Bi_15.5_ to Pb_82.9_:Bi_17.1_ (Figure 2 region I for A–C). It is noted that region II shifts to the deposition duration decreasing side, which indicates the presence of a mixed Pb:Bi phase (Bi-rich), observed from 3–10 s (for a *D_c_* = 10 mA/cm^2^) to 3–5 s (for a *D_c_* = 20 mA/cm^2^). Obviously, with a double increase in the *D_c_*, the duration required for the formation of the Bi-rich phase also decreases by half (from 10 to 5 s). After that the content of Bi in the films reaches 99.1% (Figure 2A, region II). Finally, the formation of a continuous Bi film (the concentration of Bi is 100%) without the presence of Pb with an increase in *D_c_* up to 20 mA/cm^2^ occurs with a deposition duration of more than 5 s (samples 3_20 and 4_20), in contrast to the *D_c_* = 10 mA/cm^2^, where the region III is observed after 10 s of deposition (Figure 2A,C). 

The authors of the article want to note that with deposition duration of 1 s for all series of experimental samples due to the small electrodeposited film thickness the using of EDX method can introduce errors. This may affect the quantitative analysis of the ratio of components in the film. Therefore, the best way to further study the quantitative ratio of elements in the electrodeposited films is to use a more accurate X-ray photoelectron spectroscopy (XPS) technique. Interpretation of the obtained results showed that it is necessary to pay attention to the development of further research to study the influence of the electrodeposition synthesis conditions on the quantitative ratio of the components in the thin films and studying the crystal structure, which will be represented in our future works.

### 3.3. Deposition Rate Determination 

**Figure 3:**
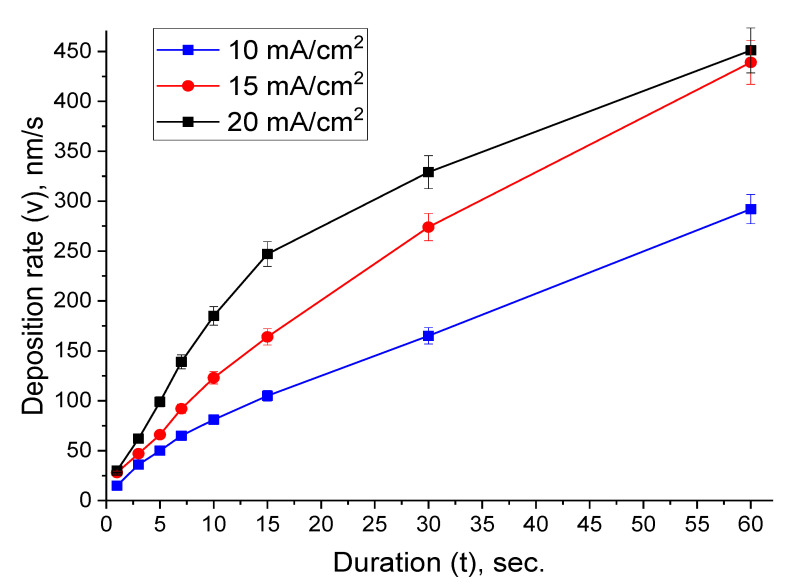
Dependence of deposition rate on duration for samples obtained with *D_c_* from 10 to 20 mA/cm^2^.

The film thickness was estimated, and the deposition rate was calculated using the data obtained from the nanoindentation method [58]. Figure 3 presents the effect of the current density on the deposition rate at various durations.

For all the studied samples, an increase in the deposition rate and thickness (Table 1) with the duration rising was observed. Moreover, the behavior of the deposition rate growth was sublinear. This tendency also was observed during electrodeposition of NiFe thin films [56]. It was noted that for current densities of 15 and 20 mA/cm^2^ with the duration of 1 s (samples 1_15 and 1_20), the deposition rate remains practically unchanged (28 and 30 nm/s), in contrast to 10 mA/cm^2^, where the rate was lower by half (15 nm/s). However, this trend changes after 1 s deposition for all ranges of current densities, which were characterized by an increase in the rate and persist up to 30 s (Figure 3). An interesting behavior of the deposition rate was observed at 60 s, where the deposition rate for *D_c_* of 15 and 20 mA/cm^2^ again differs slightly (439 and 451 nm/s). This was probably due to the fact that the *D_c_* = 20 mA/cm^2^ region approaches the boundary with the limiting current region (the diffusion plateau at the polarization curve in Figure 1), when an increase in potential does not lead to a reaction rate rise by increasing the polarization. In this case, the inhibition of the Bi film growth occurs due to the restriction by the diffusion processes. 

Thus, by varying not only the current density, but also the deposition duration (pulse deposition can be used in the future), films with a given morphology can be obtained.

### 3.4. Surface Morphology Investigation

**Figure 4:**
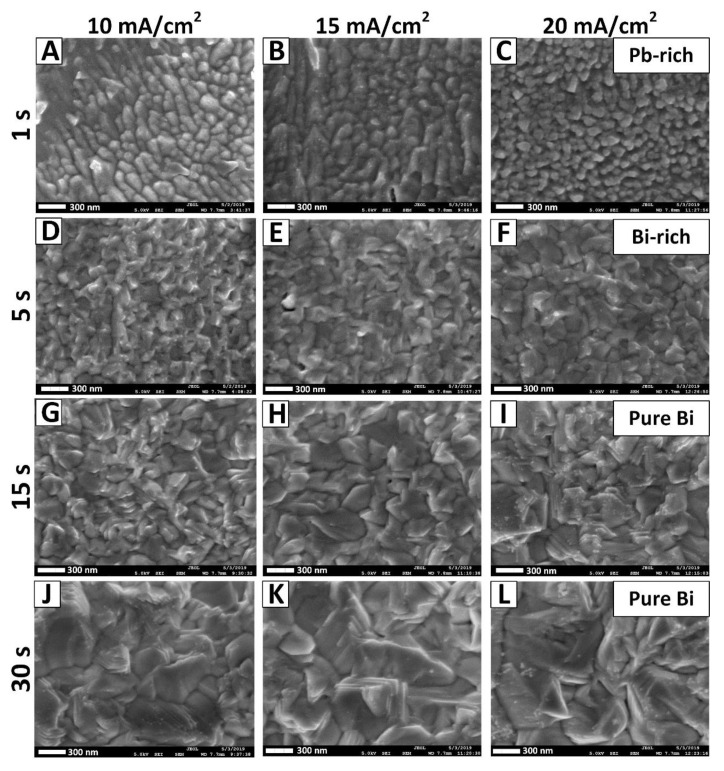
SEM images of films surface electrodeposited from PE with 10 mA/cm^2^ (**A**,**D**,**G**,**J**), 15 mA/cm^2^ (**B**,**E**,**H**,**K**), and 20 mA/cm^2^ (**C**,**F**,**I**,**L**) current density, and the deposition duration of 1 s (**A**–**C**), 5 s (**D**–**F**), 15 s (**G**–**I**), and 30 s (**J**–**L**).

The surface morphology of electrodeposited films has been studied by the SEM technique (Figure 4). It was shown that the morphology of Bi films depends on the synthesis conditions (current density, deposition duration) and on the film thickness [21]. This dependence was also found in Bi films with a thickness of 200 nm or more, obtained in tartaric acid electrolyte [9]. However, the morphology of such Bi films was different, from granular to needle-like. It can be seen from Figure 4 that with an increase in current density to 20 mA/cm^2^ and deposition duration up to 30 s, the grains morphology changes, and their average size rises (Table 1). The first stage (region I of Figure 2), which is the Pb-rich phase (samples 1_10–1_20), is characterized by a pillar-like form (Figure 4A–C). Here, the grain form represents an ellipsoid, in which the major axis is located in the parallel plane to the substrate (average grain size is ~200 nm), and the minor axis (thickness = height = 15 nm—for the 1_10 sample, for example) in the perpendicular plane to the substrate. This form depends on the synthesis conditions and the growth mechanism, as was shown in our previous study of the growth mechanisms of NiFe films [59,60]. Depending on the electrodeposition synthesis conditions, grains of an elliptical shape can form with the major axis both parallel and perpendicular to the substrate (these two cases have been studied previously in [59,60]). Then, a change in the morphology of the grains to a granular form was observed (Figure 4D–F). Finally, after 15 s of deposition, a layered granular morphology of Bi grains was observed, which was characteristic of rhombohedral Bi [52]. 

The layering of granular Bi grains occurs due to the growth texture (preferential orientation of the grains). Moreover, the layering increases with the deposition duration rising for all ranges of current densities. In order to show such a tendency toward an increase in the layering of Bi grains with the deposition duration rising, we prepared some additional samples (they are not included in Table 1 describing the main samples). 

**Figure 5:**
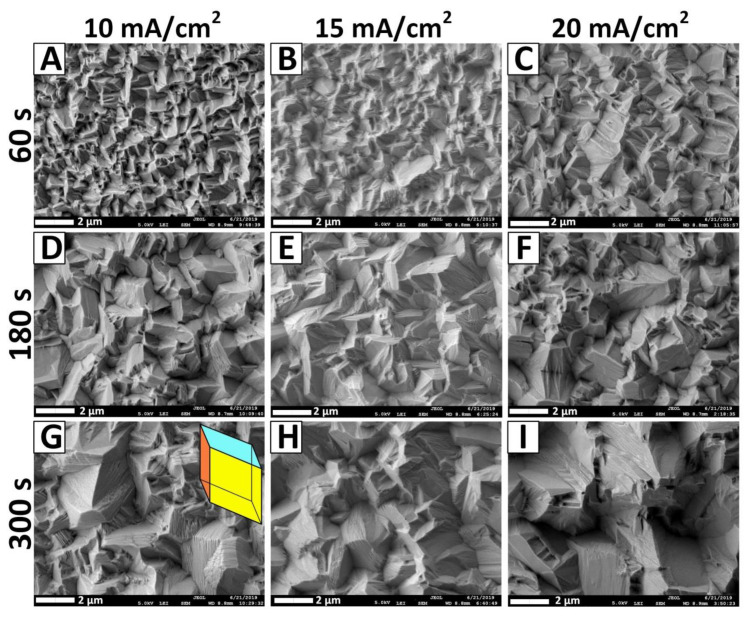
SEM images of Bi films surface electrodeposited from PE with 10 mA/cm^2^ (**A**,**D**,**G**), 15 mA/cm^2^ (**B**,**E**,**H**), and 20 mA/cm^2^ (**C**,**F**,**I**) current density, and deposition duration of 60 s (**A**–**C**), 180 s (**D**–**F**), and 300 s (**G**–**I**).

Figure 5 shows the results of the Bi samples obtained with the deposition duration of 60–300 s. Besides, not only layering occurs in Bi granular grains, but also, with an increase in the deposition duration up to 300 s, the morphology of the grains, more and more, resembled the faces of the rhombohedron (Figure 5G–I). This behavior of the Bi thin films morphology was noticed for the first time. Only the presence of a layered microstructure was noted in the thick electrodeposited Bi obtained from PE with organic additives presence and without additives with a film thickness of 600 µm [52].

The existence of such a texture is due to the presence of a direction of faster grain growth. The rate of their growth during electrodeposition is mainly determined by the current density, which in turn characterizes the amount of metal released per unit time on a unit surface of the cathode. At higher current densities, the deposition rate is greater, and conditions are created for the growth of faces with lower surface energy. An increase in current density usually leads to a rise in the rate of discharge of metal ions at the cathode without a corresponding increase in the rate of their delivery to the cathode region. As a result, the cathode region is depleted by cations, and such conditions contribute to the growth of films in the direction perpendicular to the cathode surface. Thus, as the current density increases, the crystallite orientation changes towards decreasing the texture axis indices, which was shown in our previous work [52]. Such microstructural behavior was observed in electrodeposited Ni-Co films, where the presence of texture axes was noticed depending on the pH value of the electrolyte [61] and in work [62] during the electrodeposition of Sn with a layered microstructure with the introduction of various organic additives.

Another explanation for the occurrence of texturing may be the presence of an accommodative enlargement of grains (subgrains) of the main texture component. The enlargement occurs due to their coalescence. Further, due to the coalescence of the neighboring Bi grains of the main and other texture components and with the disappearance of the boundaries separating them, the electrodeposited film is formed. This mechanism may explain the formation of the texture of a coarse-grained film, in which large grains consist of subgrain blocks. Therefore, in our case, we observe a layered textured structure with the deposition duration of more than 30 s.

**Figure 6:**
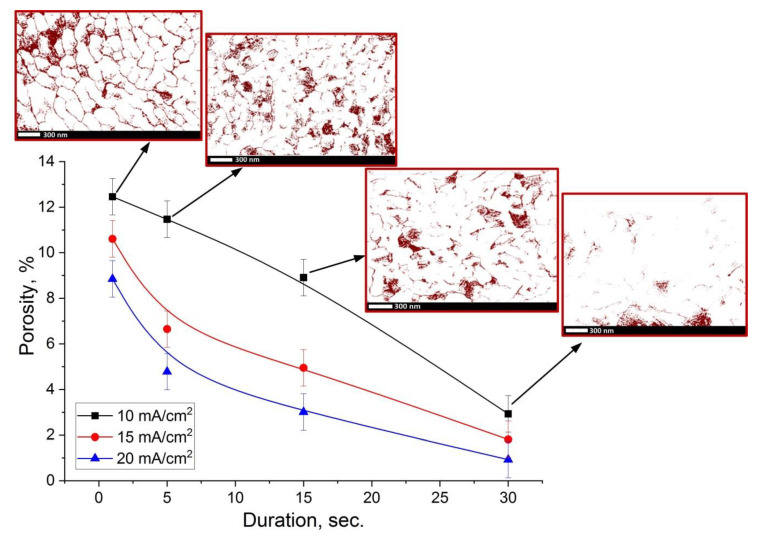
The dependence of films porosity on electrodeposition duration in PE at 10–20 mA/cm^2^ current density. The inserts show the porosity contrast images for series 3 samples (*D_c_* = 10 mA/cm^2^).

The formation of granular films during the electrodeposition in electrolytes, containing bismuth nitrate pentahydrate and nitric acid as the main components, has also been observed in work [5]. However, the grain morphology is somewhat different for our case. Here, the grains are more densely packed than in work [5], where the Bi films have high porosity and roughness. The results of the study of the dependence of the Bi films porosity on the deposition duration (1–30 s) for the main studied samples are presented in Figure 6.

The investigation of porosity contrast images obtained from SEM data showed that Bi films have a closely-packed microstructure. The porosity decreases with the increase of deposition duration for all samples. The average porosity for the Pb-rich films obtained via electrodeposition with a duration of 1 s is 10.6% (samples 1_10–1_20). However, with the deposition duration increasing, the porosity decreases, and the average value becomes 1.9% (samples 4_10–4_20). The reason for the decrease in porosity level with an increase in both the deposition duration and the current density value is the result of grain growth and filling of voids, as well as an increase in packing density. This tendency is clearly visible in Figure 6 inserts, which present the porosity contrast images. Such a closely-packed Bi microstructure contributes to the creation of materials that can be widely used in the fields where a high level of density (low porosity) is important [18,20,21,23].

### 3.5. Average Grain Size Analysis

**Figure 7:**
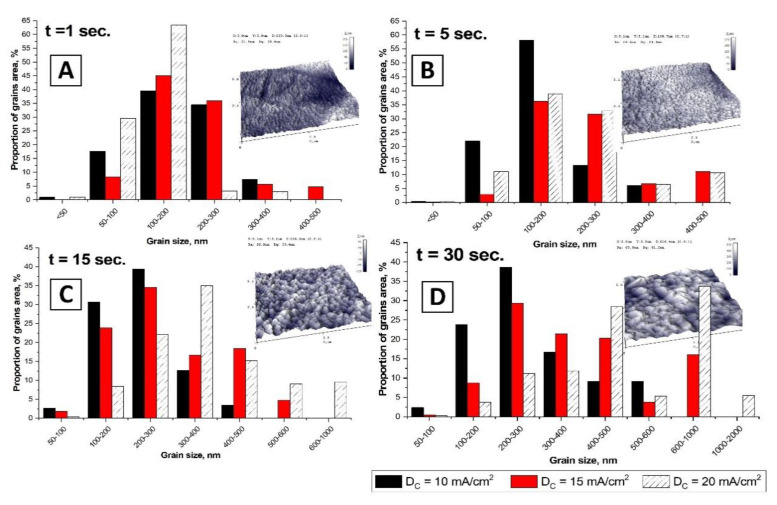
The dependence of the grain size of electrodeposited films on the duration (**A**—1 s, **B**—5 s, **С**—15 s, **D**—30 s) in a PE at current densities of 10–20 mA/cm^2^. The insert shows AFM images of the Bi films surface obtained at a current density of 10 mA/cm^2^ (scan field size is 5 µm by 5 µm).

The processing of AFM images using the Gwyddion program contributes to obtaining data on the statistical distribution of the films grain sizes dependent on the synthesis conditions [63]. The results of the statistical grain sizes distribution study on the deposition duration at a current density of 10–20 mA/cm^2^ are shown in Figure 7.

**Figure 8:**
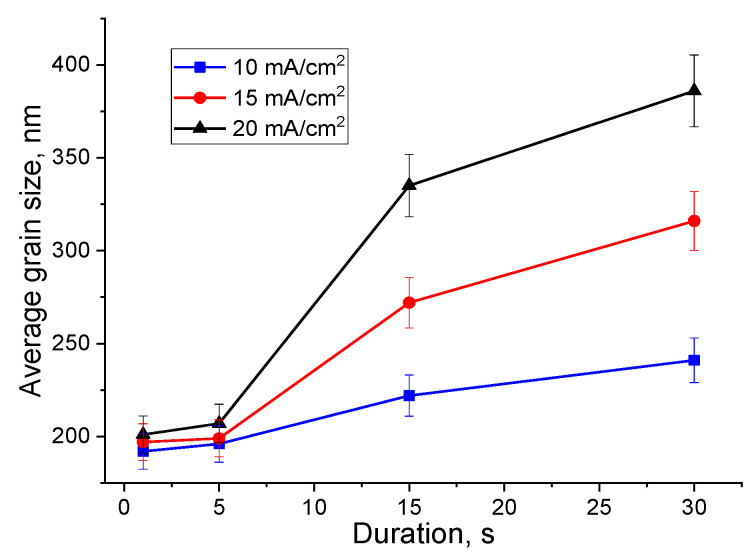
The average grain size of electrodeposited films versus deposition duration.

It can be seen from the obtained data that an increase in the deposition duration from 1 to 30 s leads to grain sizes rising. This behavior is also significantly affected by the value of current density (this is also confirmed by SEM data). For a more visual presentation of the research results we plotted the dependence of the calculated average grain size on the deposition duration (Figure 8). 

It was noted that with the deposition duration up to 5 s, a slow increase in the average grain size occurs (does not exceed 1.3 times in comparison with the deposition duration of 1 s). In addition, after 5 s this increase noticeably rises up to 30 s deposition for all series of samples. Further, an increase in current density to 20 mA/cm^2^ leads to a more abrupt rise in comparison with 10 mA/cm^2^, where the growth of the Bi average size is smoother. It was shown that an increase in the deposition duration up to 30 s with current densities 10–20 mA/cm^2^, it is possible to obtain Bi films with grain sizes 1.3–1.9 times larger than during deposition with the duration of 1 s (Table 1). The obtained results demonstrate that in the investigated current density range and deposition duration of 1–30 s, Pb:Bi and Bi films with nanosized average grain size (192–386 nm) and thickness from 15 nm to 10 µm can be obtained. In another work [20], for example, 6 µm-thick Bi films electrodeposited from nitrate electrolyte have 10 µm grains size. When citric acid and various organic additives are added to the nitrate electrolyte, it leads to the formation of dendritic, pyramidal, and branch-like grains in Bi films with grain sizes from 27 to 49 nm [32]. A significant change in grain size downward is associated with a strong adsorption effect of additives on the deposits.

### 3.6. Growth Mechanism Explanation

**Figure 9:**
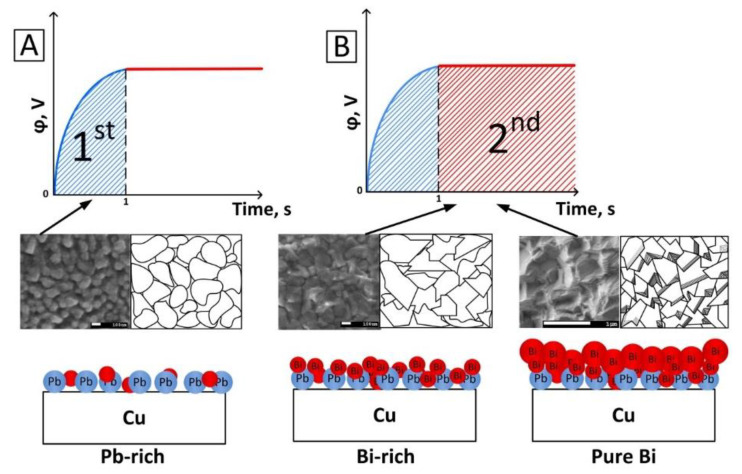
The main stages and growth mechanisms of Pb:Bi and Bi films electrodeposited from PE in the galvanostatic regime. **A**—the co-deposition of Pb and Bi (the 1st stage), **B**—Bi electrodeposition (the 2nd stage).

Summing up the results of the research, we can conclude that in the early stages of Bi electrodeposition (at short electrodeposition duration) from PE, deposition is carried out in two main stages (Figure 9). 

At the moment of switching on the electrochemical cell (turning on the current source), a sharp in-rush current occurs in the system, which exists until it stabilizes at a given value. During the electrodeposition in the galvanostatic regime, a change in potential occurs in order to adjust it to a given current value. It is known that the formation and growth of a metal film on the cathode surface is a potential-dependent process [64]. During this time (1st stage in Figure 9A), which, apparently, is of the order of 1 s (samples 1_10–1_20), the co-deposition of Pb and Bi occurs (formation of the Pb-rich phase). However, this process is also associated with a relatively slow growth of Bi grains, which is confirmed by the EDX results (Figure 2). Under the electrochemical dissolution of Bi anodes, consisting of a PbO_2_ including alloy, the lead (IV) oxide dissolves in the perchloric acid and further dissociates into the solution by the following reaction:

(3) $$\mathrm{Pb}{\left({\mathrm{ClO}}_{4}\right)}_{4}\;\to {\mathrm{Pb}}^{4+}+4{\mathrm{ClO}}_{4}^{-}$$

Then, the dissociated Pb^4+^ ions are reduced at the cathode:

(4) $${\mathrm{Pb}}^{4+}+4e^{-}\;\to {\mathrm{Pb}}^{0}$$

The standard electrochemical potential of Pb^4+^ → Pb^0^ reduction is about 0.77 V. Therefore, at the first moment of time, Pb is mainly reduced at the cathode, since its potential is more positive relative to the Bi potential. In PE, the standard Bi electrode potential is 0.32 V. The process of bismuth reduction at the cathode comes from bismuth perchlorate:

(5) $$\mathrm{Bi}{\left({\mathrm{ClO}}_{4}\right)}_{3}\;\to {\mathrm{Bi}}^{3+}+{\;\mathrm{ClO}}_{4}^{-}$$

The second stage of Bi electrodeposition is associated with a current stabilization when the current has reached its predetermined value (2nd stage in Figure 9B). In this case, the predominant growth of Bi grains occurs. According to the EDX data (Figure 2), three main regions of film growth during electrodeposition can be seen. However, in the description of the mechanism of film growth, we combined regions II and III (Figure 2A–C), since both are associated with Bi growth. In region II (Figure 2A–C), the presence of a Bi-rich phase with a low Pb content (up to 1.9%) was observed. The duration of this region is decreased with current density increasing. This process is associated with the Bi grains growth on the surface of Pb grains, which occurs faster with current density rising (Figure 9B). The surface morphology, in this case, begins to resemble Bi grains more and more and, with an increase in the deposition duration, only Bi grows (samples 3_10–3_20 and 4_10–4_20). This is evidenced by the fact that at a current density of 20 mA/cm^2^ (sample 2_20), region II ends at 5 s (Figure 2C). Over time, the deposition rate of Bi increases, and we do not detect Pb in the films.

## 4. Conclusions

The nanocrystalline Bi films have been electrochemically deposited onto the Cu substrate, using a galvanostatic regime with various duration (1–30 s) and current density of 10–20 mA/cm^2^. We examined the morphology, microstructure, and chemical composition features at the initial stages of the growth process of the films formed in perchlorate electrolyte. The EDX analysis showed that in the short duration (1 s) of electrodeposition the Pb-rich phase in Bi films arises. The mechanism of co-deposition of Pb and Bi in films is explained. It has been demonstrated that it is possible to deposit Pb films using Bi rods with a PbO_2_ impurity on the Cu substrate under synthesis conditions control. We investigated the impact of deposition duration, current density, and thickness on the deposition rate, average grain size, and morphology of Bi films. While the current density determined the deposition rate as expected, it had an influence on films thickness and grain size. Current density-dependent film thickness was the primary determinant of the grain size, showing an approximately sublinear relationship. The morphology of films also depends on such key parameters as duration and current density. Due to an increase in current density, up to 20 mA/cm^2^ and deposition duration up to 30 s, the grains morphology changes and their average size rises. It has been shown that in the first stage of deposition the Pb-rich phase is characterized by a pillar-like form that changes to granular and layered granular form due to the processes of Bi grains growth and their coalescence. The investigation of porosity contrast images obtained from the SEM data showed that Bi films have a closely-packed microstructure. The porosity decreases with increasing the deposition duration for all samples. A new approach of Bi electrodeposition using perchlorate electrolyte is proposed for the formation of high-quality, dense nanocrystalline films with controlled morphology. It will allow the formation of promising Bi films for use in many practical applications, especially in areas where high-density value is important, for example, in radiation materials science and nuclear technology.

## Figures and Tables

**Table 1:** The description of synthesis conditions and parameters of the main experimental samples.

Series	Sample No.	Current Density (*D_c_*), mA/cm^2^	Deposition Duration (*t*), s	Deposition Rate (*v*), ~nm/s	Thickness (*h*), nm	Composition of Pb:Bi (*C*), %	Porosity (*p*), %	Average Grain Size, nm
1	1_10	10	1	15	15	84.5:15.5	12.5	192
	2_10		5	50	252	0.9:99.1	11.5	196
	3_10		15	105	1575	0:100	8.4	222
	4_10		30	165	4950	0:100	2.9	241
2	1_15	15	1	28	28	83.3:16.7	10.6	197
	2_15		5	66	329	1.4:98.6	6.7	199
	3_15		15	164	2465	0:100	5.0	272
	4_15		30	274	8223	0:100	1.8	316
3	1_20	20	1	30	30	82.9:17.1	8.4	201
	2_20		5	99	445	1.2:98.8	4.8	207
	3_20		15	247	3704	0:100	3.0	335
	4_20		30	329	9879	0:100	0.9	386
